# Analysis of Infectious Diseases in Himi City, Japan, During the Noto Earthquake in 2024 Amid the Ongoing COVID-19 Pandemic

**DOI:** 10.7759/cureus.76689

**Published:** 2024-12-31

**Authors:** Yuko Tanihata, Kinue Takebayashi, Hideto Kitagawa, Masaharu Iguchi, Yoshitsugu Iinuma, Takuya Sakamoto, Tomoyuki Ushimoto, Yuji Kasamaki, Tsugiyasu Kanda

**Affiliations:** 1 Department of Patient Safety Management, Kanazawa Medical University Himi Municipal Hospital, Himi, JPN; 2 Department of Respiratory Medicine, Kanazawa Medical University, Uchinada, JPN; 3 Department of Infectious Diseases, Kanazawa Medical University, Uchinada, JPN; 4 Department of Pharmacology, Kanazawa Medical University, Uchinada, JPN; 5 Department of Emergency Medicine, Kanazawa Medical University, Uchinada, JPN; 6 Department of Community Medicine, Kanazawa Medical University Himi Municipal Hospital, Himi, JPN

**Keywords:** covid-19, earthquake, evacuation center, influenza, outbrake

## Abstract

Introduction: Earthquakes are among the most striking natural phenomena, with a very high potential to set off a chain of events, including casualties and injuries, substantially impacting public health. In particular, earthquakes can trigger outbreaks and epidemics of infectious diseases during the post-earthquake period. Accordingly, we analyzed infectious diseases in Himi City during the Noto earthquake in January 2024, a period during which COVID-19 infections were also prevalent.

Materials and methods: A cross-sectional study was conducted from January 1, 2024, to January 14, 2024, to obtain precise, timely data on public health and estimate the short-term occurrence of infectious diseases among disaster victims after the Noto earthquake. We studied the medical records of all patients who visited the emergency department of Kanazawa Medical University Himi Municipal Hospital, located near the Noto earthquake. Of the 411 participants evaluated, 218 patients (53%) were males and 193 (47%) were females. Of the 411 people, 275 (67%) had an infectious disease. We investigated specific COVID-19 infection locations where individuals may have been infected, including primary evacuation centers, and examined the number of cases on each day of consultation.

Results: Infections at evacuation centers peaked on January 4 and 5, 2024. Infections at primary evacuation centers occurred on January 9, 2024.

Conclusion: A combined pandemic and earthquake disaster may worsen disease severity. To control the risk factors of infectious diseases, primary shelters should improve our consideration of the effects of interventions and prepare for potential infectious disease outbreaks.

## Introduction

Earthquakes are one of the most striking natural phenomena that can lead to a cascade of events resulting in casualties and injuries, thereby substantially impacting public health [1]. Earthquakes trigger the emergence of sporadic cases, outbreaks, and epidemics of infectious diseases during the post-earthquake period, earthquakes of magnitude ≥ 5.6 MW. Upper respiratory tract infections are frequent in the short term [2]. Most earthquake-affected individuals live in overloaded evacuation shelters with insufficient air ventilation, unsafe drinking water, and reduced personal hygiene, among the thinkable predisposing factors for contracting respiratory infectious diseases [3]. To control the risk factors of infectious diseases, partaking data on the status and control of infectious diseases and related expert groups should be prioritized to prepare for potential outbreaks of infectious diseases [4].

Japan is one of the most earthquake-prone countries worldwide. Notably, respiratory and gastrointestinal (GI) diseases have been predominantly recorded among those affected by earthquakes [5]. On January 1, 2024, the Noto Peninsula earthquake struck Himi City, Toyama Prefecture, with a seismic intensity of ≥5, and a tsunami warning was issued approximately 10 minutes later. Approximately 13.9% of the population (approximately 6,000 individuals) spent the night in evacuation centers on higher ground. The water supply of 14,000 households was cut off for two weeks from January 1, 2024, owing to a broken water pipe caused by an earthquake [6]. In this study, we aimed to analyze the occurrence and dynamics of infectious diseases in Himi City during the Noto earthquake in January 2024, a period marked by the widespread prevalence of Coronavirus disease 2019 (COVID-19). The analysis investigated the compounded effects of the earthquake and the pandemic, with a particular focus on the transmission of COVID-19 and other infectious diseases within evacuation centers and the broader community. This dual-disaster scenario offered valuable insights into the challenges of managing infectious disease outbreaks in post-disaster settings.

## Materials and methods

**Figure 1:**
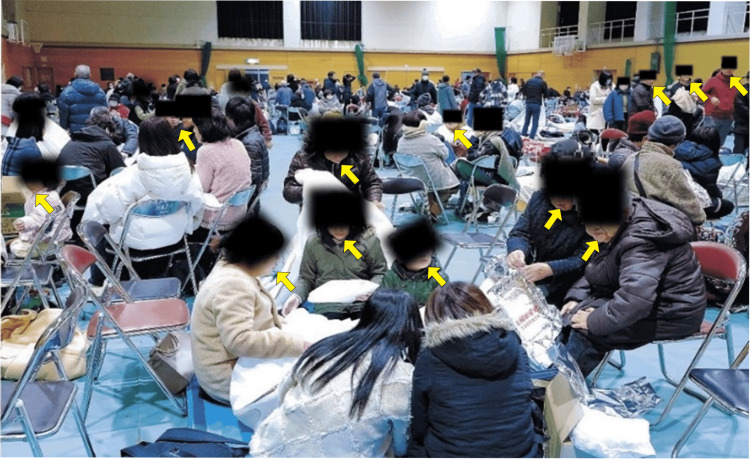
Citizens evacuating to shelters in Himi City, Japan after the January 1, 2024, earthquake A large group of citizens gathered in a gymnasium that was converted into an evacuation shelter after the earthquake on January 1, 2024. As shown in the image, many evacuees are sitting in proximity without wearing masks (indicated by yellow arrows). Approximately 14.0% of the city's population evacuated to shelters following a tsunami warning. Credit: Permission to reproduce this image has been obtained from the copyright holder, Toyama Shimbun.

As shown in Figure 1, several citizens did not wear protective masks despite the crowded shelter conditions. On January 2, the number of evacuees who returned home plummeted to 0.8% (approximately 350 individuals). Immediately thereafter, the water supply to the city was cut off, and the number of patients in the hospital's fever outpatient clinic increased rapidly.

We conducted a descriptive observational study on a consecutive case series of patients with respiratory and GI tract infections. Of the 411 participants evaluated, 218 patients (53%) were males and 193 (47%) were females. About 275 (67%) individuals had infected diseases. The study group comprised patients diagnosed with COVID-19, influenza infection, upper respiratory inflammation, GI tract infection, and pneumonia who visited the emergency department of Kanazawa Medical University Himi Municipal Hospital from January 1, 2024 to January 14, 2024.

Diagnosis of infectious diseases

COVID-19 was diagnosed by the kit of Standard Q COVID-19 Ag (SD Biosensor, South Korea). Influenza was diagnosed by the kit of Quick Chaser Flu A, B (Mizuho Medy Co., Ltd., Japan). Other infections were diagnosed comprehensively by clinical symptoms, blood tests, and chest X-rays.

Ethical statement

This study was approved by the Ethics Committee of Kanazawa Medical University Himi Municipal Hospital (approval number: 231). The study protocol was conducted in accordance with the principles of the Declaration of Helsinki.

Statistical analysis

Statistical analysis was performed using GraphPad Prism (Version 9.4.1; GraphPad Software Inc., San Francisco, USA). The results are expressed as the mean ± standard deviation. Statistical significance was defined as p < 0.05, indicating significance.

## Results

Herein, we analyzed the data of patients aged greater than or equal to 15 years with respiratory and GI tract infections extracted from the day of the earthquake on January 1, 2024. Children under the age of 15 were excluded from this study. We reviewed the medical records of all patients who visited the emergency department of Kanazawa Medical University Himi Municipal Hospital in Himi City, close to the Noto earthquake. A total of 411 subjects were evaluated, and 256 were diagnosed with infections. Of them, 136 (53%) were males and 120 (47%) were females. Additionally, we investigated locations where individuals may have contracted COVID-19 and examined the number of cases on each consultation day.

**Figure 2:**
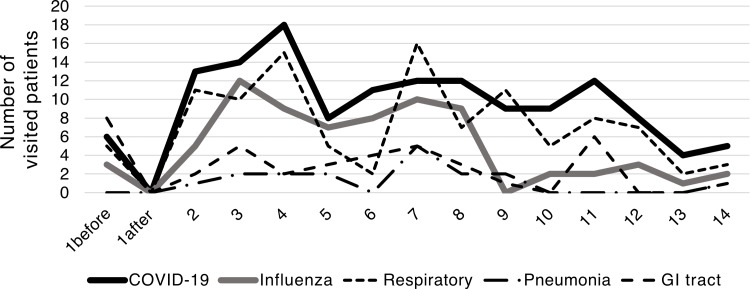
Infectious disease just after earthquake in January 2024 “1 before” means the time before the earthquake on January 1st; “1 after” means the time after the earthquake on January 1st.

**Figure 3:**
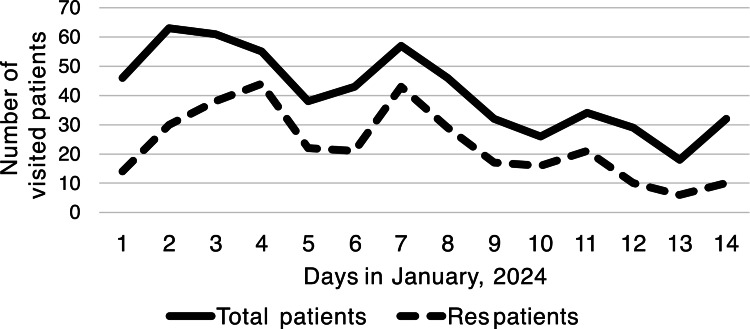
Number of total patients and respiratory and GI tract infection patients (Res patients) admitted to the emergency room from January 1 to 14, 2024

**Figure 4:**
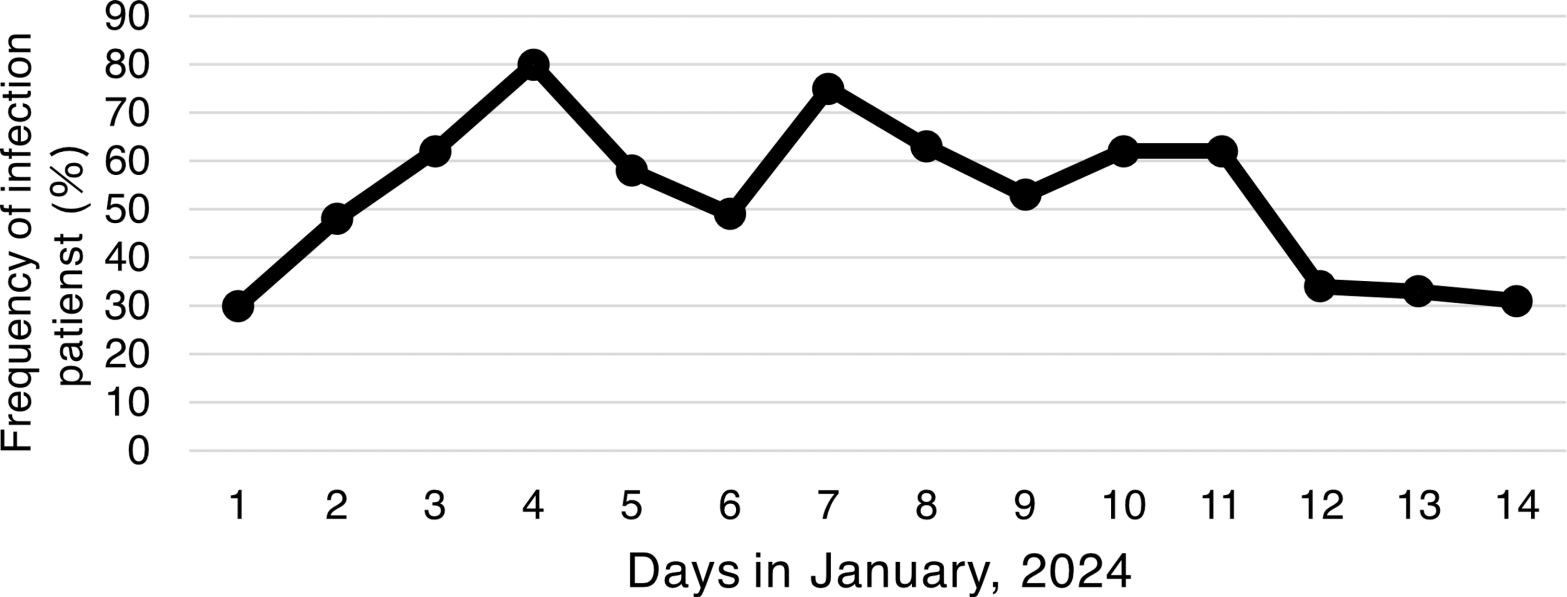
Frequency of respiratory and GI tract infection patients per total patients

There was no difference in age or sex between the five groups: COVID-19, influenza infection, upper respiratory tract inflammation, and GI tract infections; only older individuals developed pneumonia (Table 1). The number of COVID-19 cases peaked on January 4, while influenza infections peaked on January 3, one day earlier than COVID-19. The upper respiratory tract infection occurred on the same day as that of COVID-19. The highest number of pneumonia cases was observed on January 7. The incidence of GI tract disorders peaked on January 3 and 7. Infections at evacuation centers peaked on January 4 and 5, with no infections detected beyond January 8 (Figures 2-3). The proportion of emergency patients with infectious diseases peaked on January 4 (Figure 4).

**Table 1:** Age and sex distributions of patients with infections in Himi Municipal Hospital *p < 0.05 vs. COVID-19

Observation period (January 2, 2024 to January 7, 2024)					
Types of infection	COVID-19 infection	Influenza virus infection	Upper respiratory inflammation	Gastrointestinal tract infection	Pneumonia
Age (years)	55.1 ± 19	48.0 ± 17	45.2 ± 20	53.1 ± 21	81 ± 11*
No of patients (count)	71	43	41	7	6
Male	38	26	22	2	2
Female	33	17	19	5	4
Observation period (January 8, 2024 to January 14, 2024)					
Age (years)	57.9 ± 14	55 ± 16	54.2 ± 17	91	71
No of patients (count)	57	18	30	1	1
Male	25	10	19	0	1
Female	32	8	11	1	0

**Figure 5:**
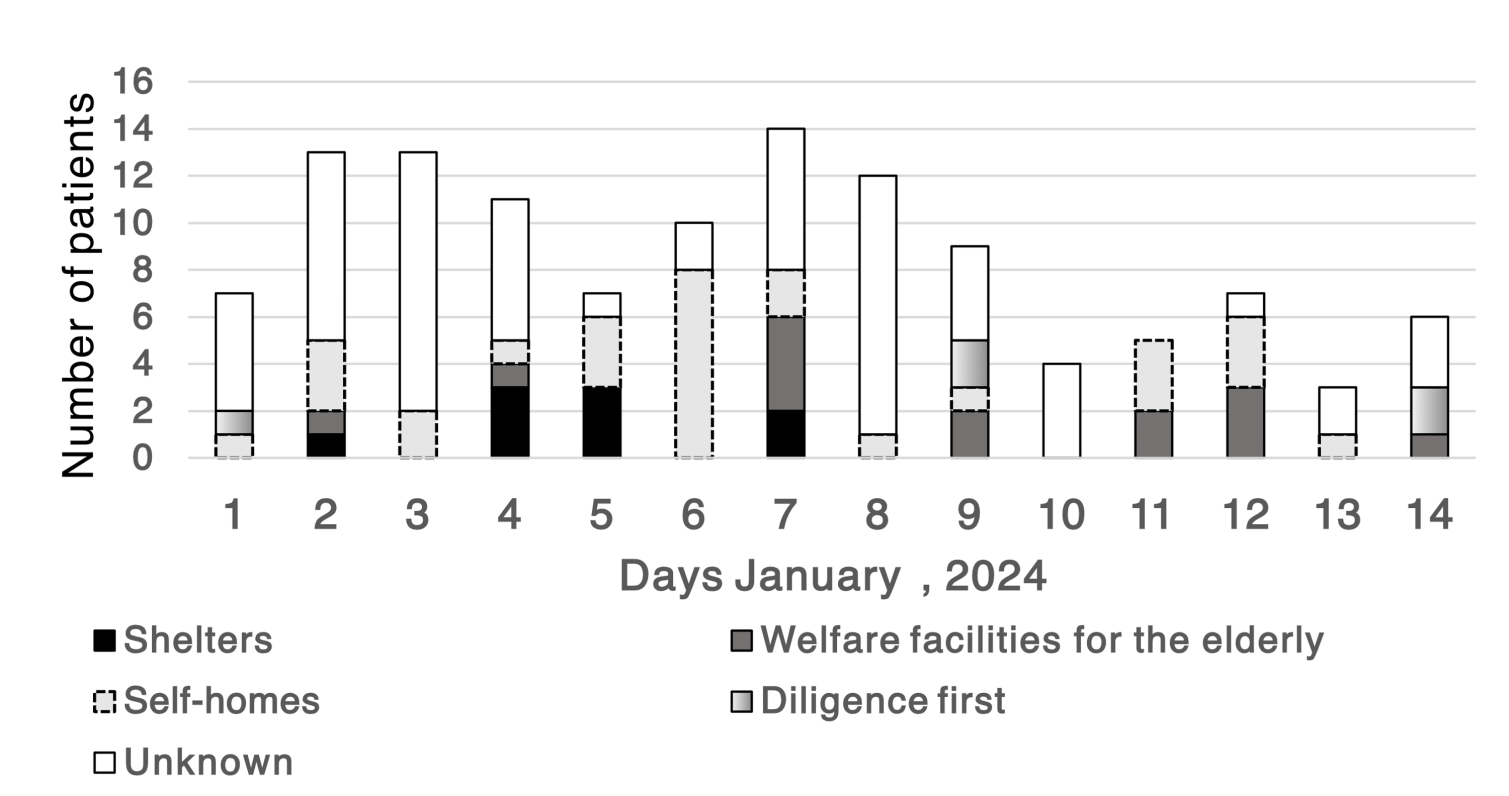
Infected places in patients with COVID-19

The highest number of infections was detected in nursing homes on January 7. The highest number of infections occurred at home on January 6, although the number of infected individuals increased daily (Figure 5). Infections at primary shelters occurred from January 9.

## Discussion

In this study, we found that a markedly high number of patients with COVID-19, influenza infection, upper respiratory tract inflammation, and GI tract disorders visited the emergency department of Kanazawa Medical University Himi Municipal Hospital from January 2 to January 14, 2024, immediately after the earthquake on January 1, 2024. The increase in infections following the earthquake may be due to the fact that several victims were living in evacuation centers. The combined disaster of an earthquake in the midst of a pandemic highlighted the importance of disaster prevention measures and the appropriate response of medical personnel at evacuation centers and nursing homes.

The strength of this research is that it is an infectious disease that occurs during natural disasters such as earthquakes. COVID-19 and influenza showed that infections spread immediately after a disaster. It was proved that it is necessary to prepare in advance for infection prevention measures at evacuation centers.

Herein, more than 10% of the population spent the night in evacuation centers on higher ground. As part of the infection control measures at the evacuation center, hand hygiene products were installed at the entrance of the evacuation center; however, the use of protective masks was left to one's judgment. The city's disaster prevention plan included masks, disinfectants, and thermometers. However, the disaster occurred on New Year's Day, and the facility was locked, and there were no staff present; hence, it is unclear whether items were used. From the news photos taken on the day of the disaster (Figure 1), the mask-wearing rate was low, suggesting that the infection had spread. The importance of wearing protective masks, their relative usefulness, and the concomitant procedures of mask adherence would be highly relevant to the measurement of effectiveness, especially among older individuals and young children. Hand hygiene can reduce the burden of respiratory illness according to standard Cochrane methodological procedures [7].

The Great Hanshin-Awaji Earthquake struck the southern part of Hyogo Prefecture in 1995. The increase in the number of hospitalized patients within the first month was mainly attributed to the rise in the number of patients with respiratory diseases. The number of patients with respiratory diseases increased by approximately 4.5 times immediately after the earthquake [8]. Two current major natural disasters, the Great East Japan Earthquake and the Kumamoto Earthquake in 2011 and 2016, respectively, resulted in a large number of deaths, along with several individuals developing infectious diseases in evacuation centers and shelters [9]. Considering the recent Noto earthquake, the Disaster Medical Support Team (DMAT) nationwide cooperated and dispatched a series of DMATs to support medical and public health needs in Wajima City, which included damages, non-transmissible diseases, infectious diseases, psychological health issues, and maternal and child health issues [10]. Crowding in evacuation shelters enlarged the risk of infectious disease outbreaks, including COVID-19. The infectious disease frequency reportedly increased among emigrants at shelters after the Great Eastern Japan earthquake and tsunami in 2011 [11].

Infections following earthquakes can occur due to living conditions in evacuation centers. Overcrowding, insufficient ventilation, dirty water, and poor personal hygiene have been identified as predisposing factors. A large number of citizens tend to rush to evacuation centers owing to the collapse of houses and tsunami warnings. According to a city survey, 14.0% of all citizens gathered at an evacuation center at 9 p.m. on January 1, 2024. Tsunami warnings were replaced with warnings, and most citizens returned home. Several individuals at evacuation centers on January 1, 2024, had COVID-19 on January 3 and 4, 2024. This time-lapse coincides with the incubation period of the Omicron variant. The COVID-19 pandemic, in conjunction with the earthquake, reportedly worsens disease severity and psychological disorders [12]. Long-term negative effects may continue under earthquakes when compared with the COVID-19 pandemic [13].

The incubation period of COVID-19 (approximately 6.4 days) is longer than that of influenza (3.4 days) [14]. According to the current analysis, COVID-19 infections peaked later than influenza infections. In terms of severity and silver bullets, COVID-19 should be carefully considered; therefore, in the case of infectious diseases during earthquakes, attention should be paid four days after occurrence. Given that pneumonia cases occur from day 7, it is important to prevent infections in older individuals. Considering the limitations of the current study, it included a small city with a population of 40,000, which was not the earthquake center, and the survey was limited to a single facility. To date, no investigations other than those exploring infectious diseases have been conducted. However, it is important to underscore the occurrence of diseases in the vicinity of earthquakes.

During the COVID-19 pandemic, earthquakes occurred in Albania in 2019 and in Croatia, Mexico, and Utah in 2020, which exposed obstacles such as the fragility of healthcare systems, the collapse of COVID-19 protection protocols, and the psychological pressure of double natural disasters [15]. Measures include strengthening the infection prevention system, providing public economic support, and dispatching medical personnel to evacuation centers.

The limitations of this study include the small number of patients and the limited capacity of the emergency hospital. Wide disaster areas are essential for examining infected patients. Further investigations are needed in the future.

## Conclusions

The combined natural disasters of the 2024 Noto Peninsula earthquake and the COVID-19 pandemic highlighted the importance of disaster prevention and how to prepare medical personnel at evacuation centers and nursing homes. To control the threat factors of infectious diseases, the management of infectious diseases in regions with community and healthcare providers, including primary shelters, warrants an improved consideration of the effects of involvement and preparation for conceivable infectious disease outbreaks.
